# Child Acute Malnutrition and Mortality in Populations Affected by Displacement in the Horn of Africa, 1997–2009

**DOI:** 10.3390/ijerph9030791

**Published:** 2012-03-06

**Authors:** John B. Mason, Jessica M. White, Linda Heron, Jennifer Carter, Caroline Wilkinson, Paul Spiegel

**Affiliations:** 1 School of Public Health and Tropical Medicine, Tulane University, 1440 Canal Street, New Orleans, LA 70118, USA; Email: jmwhite25@gmail.com (J.M.W.); linda_heron@yahoo.com (L.H.); jenncart@gmail.com (J.C.); 2 United Nations High Commissioner for Refugees, Case Postale 2500, CH-1211 Geneva 2 Dépôt, Switzerland; Email: wilkinso@unhcr.org (C.W.); Spiegel@unhcr.org (P.S.)

**Keywords:** malnutrition, refugees, humanitarian assistance, Africa, drought

## Abstract

Drought and conflict in the Horn of Africa are causing population displacement, increasing risks of child mortality and malnutrition. Humanitarian agencies are trying to mitigate the impact, with limited resources. Data from previous years may help guide decisions. Trends in different populations affected by displacement (1997–2009) were analyzed to investigate: (1) how elevated malnutrition and mortality were among displaced compared to host populations; (2) whether the mortality/malnutrition relation changed through time; and (3) how useful is malnutrition in identifying high mortality situations. Under-five mortality rates (usually from 90-day recall, as deaths/10,000/day: U5MR) and global acute malnutrition (wasting prevalences, < −2SDs of references plus edema: GAM) were extracted from reports of 1,175 surveys carried out between 1997–2009 in the Horn of Africa; these outcome indicators were analyzed by livelihood (pastoral, agricultural) and by displacement status (refugee/internally displaced, local resident/host population, mixed); associations between these indicators were examined, stratifying by status. Patterns of GAM and U5MR plotted over time by country and livelihood clarified trends and showed substantial correspondence. Over the period GAM was steady but U5MR generally fell by nearly half. Average U5MR was similar overall between displaced and local residents. GAM was double on average for pastoralists compared with agriculturalists (17% *vs*. 8%), but was not different between displaced and local populations. Agricultural populations showed increased U5MR when displaced, in contrast to pastoralist. U5MR rose sharply with increasing GAM, at different GAM thresholds depending on livelihood. Higher GAM cut-points for pastoralists than agriculturalists would better predict elevated U5MR (1/10,000/day) or emergency levels (2/10,000/day) in the Horn of Africa; cut-points of 20–25% GAM in pastoral populations and 10–15% GAM in agriculturalists are suggested. The GAM cut-points in current use do not vary by livelihood, and this needs to be changed, tailoring cut points to livelihood groups, to better identify priorities for intervention. This could help to prioritize limited resources in the current situation of food insecurity and save lives.

## 1. Introduction

Given the recent severe food insecurity in the Horn of Africa [1,2] and the tens of thousands of refugees streaming into camps in Djibouti, Ethiopia and Kenya–with high mortality and acute malnutrition rates not seen for over a decade—a study of earlier (1997–2009) malnutrition and child mortality levels and trends in the region is timely and informative. 

UNHCR’s Global Report 2005 [3] (*i.e.*, with data from around the mid-point of the period studied here) for the Horn of Africa estimates that at that time there were 750,000 refugees and 1,250,000 other persons of concern plus 840,000 internally displaced people (IDPs) and 150,000 refugees in Sudan; all persons involuntarily migrated from their homes and livelihood. Thus approximately 3 million people were displaced, as refugees or IDPs. The causes are complex, but particularly include drought and conflict. Some of these refugees and IDPs (hereafter referred to as displaced persons) were housed in camps—many for years—run by United Nations (UN) agencies with non-governmental organization (NGO) partners. They received varying levels of humanitarian assistance, mainly medical, food aid, shelter, protection and core relief items. Others merged with the local populations, sometimes also having access to humanitarian assistance. Under these conditions, especially in children, acute malnutrition and elevated mortality rates were of particular concern.

We examined the results of 1,175 small-scale surveys carried out during 1997–2009 that assessed the extent and severity of acute malnutrition and child mortality. Analyses are secondary, based primarily on the results available from the surveys themselves. Surveys typically sampled approximately 900 households often using a 30 cluster × 30 household design, and followed the methods given in the ‘SMART Handbook’ [4]. Survey design and implementation were not under our control, results being taken from the published reports as compiled by UN agencies (Standing Committee on Nutrition (SCN) and High Commissioner for Refugees (UNHCR)) and the Centre for Research on the Epidemiology of Disasters (CRED). The available survey results were compiled into a database to address questions of operational relevance for the Horn of Africa: (1) how elevated were malnutrition and mortality among displaced populations compared with host populations and what were their trends through time; (2) whether the relation between malnutrition and child mortality changed through time, and (3) how useful malnutrition prevalence was, or could be, in identifying populations with elevated mortality as priority for assistance. Growth patterns of children in the Horn of Africa vary greatly depending on whether the population’s livelihood is agricultural (*i.e.*, crop growing) or pastoral (*i.e.*, depending on cattle) [5]. For instance, comparing Ugandan (mainly agricultural) and Somali (pastoral) pre-school children under their normal conditions using national surveys, the global acute malnutrition (GAM) prevalences were approximately 5% and nearly 20%, respectively. Moreover, comparative height growth occurred in the other direction (*i.e.*, Somali children grow faster in length, so that for example by 1 year Ugandan child stunting rates are about 40%, and Somali 25%). These effects balance each other in the sense that underweight prevalences are similar (*i.e.*, 30% and 25%, respectively) [6]. However, small-scale surveys do not usually determine sampled children’s ages, so the measure of malnutrition depends primarily on weight-for-height (*i.e.*, wasting) [Global Acute Malnutrition (GAM) is the usual indicator, combining wasting prevalences (<–2SDs weight-for-age); since oedema is usually 1% or less, GAM and wasting are nearly identical, and for the present purposes (since oedema prevalences were not recorded) treated as the same)]. Thus, both the relation of acute malnutrition (when measured as GAM) to mortality, and hence the interpretation of GAM prevalences in considering mortality risk, need to take into account livelihoods, specifically agricultural or pastoralist. An objective was to examine the implications of taking livelihood into account in this context. The survey methods and results, while following standard procedures, did not always include U5MR (33% of surveys in the database did not estimate U5MR). One aim therefore was to indicate likely U5MR levels from GAM estimates, for when U5MR is not measured, and to contribute to the interpretation when both are.

Interpretation of survey results in terms of urgency of need for assistance depends on comparisons with cut-points, which presently do not vary among populations. For example, for child GAMs, the World Health Organization (WHO) recommends that a GAM prevalence of 10–15% is ‘serious’, and above 15% ‘critical’, for all populations [7]. The UN Standing Committee on Nutrition (SCN) [8] suggests that over 10% wasting prevalence is a serious situation. Under-5 mortality (U5MR) of >2/10,000/day is taken as an alert level indicating an emergency and urgent need for action [8,9,10], and 1/10,000/day is a warning level. Similar cut-points for U5MR and GAM are used for Somali data by FAO [11]. However, the relation between malnutrition (GAM) and mortality has not been used before to assess the implications of different levels of these indicators; rather they have been used in tandem (e.g., as in [11]) together with other indicators. 

Previous studies on populations less affected by displacement demonstrated that the relation between mortality and GAM varied by livelihood, suggesting variable GAM cut-points for different populations ranging from 8% to 20% GAM for agriculturalists *versus* pastoralists, respectively [6]. 

This study aims to improve the understanding of the practical interpretation of malnutrition levels in relation to mortality risk, from analysis of routine data usually available in emergencies. While further information—such as length of time displaced or of receipt of humanitarian assistance—would be useful, these were not available, as is often the case in actual decision-making. We aim to present a pragmatic analysis based on the data available, having established the most extensive compilation of survey results of this type yet accumulated, as far as is known.

## 2. Methods

Data were assembled by extracting survey results (aggregated prevalences and mortality estimates) from the UNSCN NICS (Nutrition in Crisis Situations) database [12], plus additional survey results from the United Nations High Commissioner for Refugees’ (UNHCR) health information system (HIS) and the Centre for Research on the Epidemiology of Disasters (CRED) [13]. Data from Ethiopia, Kenya, Sudan, and Uganda were selected; initially data from Somalia were included, however the data were of more uncertain quality and preliminary results showed more consistency from the other four countries, thus the Somali results are not reported here. The distribution of sources of survey results was 85% from NICS, 1% from UNHCR’s HIS, and 14% from CRED. Duplicates were eliminated, giving valid cases of 1,175 for GAM and 782 for U5MR. Sample sizes were recorded available for 44% of the surveys, and of these 75% were between 700 and 1000. All U5MR cases also had GAM recorded. GAM and U5MR results were obtained by surveys carried out by NGOs or UN agencies, almost always following a sampling procedure similar to the 30 cluster by 30 household design [14]. The original data are not included in the databases, are not readily available, and could not be accessed for this study.

GAM is defined as percentage of the children sampled in each survey of less than minus 2 standard deviations of reference standards, plus prevalence of oedema (usually a fraction of wasting prevalence). When prevalences were reported based on NCHS standards, these were transformed to the equivalent using WHO standards using the WHO algorithm [14] (neglecting oedema). U5MR was estimated generally using a 90-day recall period, and is given as deaths/10,000/day. Extreme values were excluded to avoid their undue influence on the results as follows. Values greater than 50% GAM prevalence were excluded (8 cases: >+4SDs). A cut-point for U5MR of greater than 7 (>3SDs; 23 cases) was used. Otherwise all data from 1997 to 2009 were included. 

The displacement status of the populations surveyed is included in the NICS and other databases from where the data were taken, and was copied unchanged. In the data used, 14% were from populations defined as refugees, 9% IDPs, 59% were defined as resident (*i.e.*, local surrounding population), and 18% mixed displaced and resident. The refugee and IDP data were combined into a category called displaced to increase sample size. The distribution of data was: Ethiopia 513 (44%), Kenya 125 (11%), Sudan 436 (37%), and Uganda 101 (9%).

Livelihood (pastoralist and agriculturalist) was not recorded in the survey results, but was assessed from where the population surveyed was located, from the livelihood (and ecology) of the area, recorded at the district level or equivalent. In Ethiopia pastoralists was defined as in Afar and Somali regions, and agriculturalists in others; in Uganda pastoralists were defined as in Karamoja. In Kenya all surveys were in pastoral areas of Northeast or Rift Valley provinces. In Sudan, surveys were from predominantly pastoral areas, and were assigned to the pastoral category.

Data was analyzed using the Statistical Package for Social Sciences (SPSS) version 16 and Microsoft Excel. Results in Table 1 and Table 2 were calculated using SPSS ‘compare means’, ANOVA, and non-parametric tests (Mann-Whitney and Kruskall-Wallis) as discussed below. GAM and U5MR were categorized to calculate sensitivity and specificity, by cross tabulation, as shown in Table 3 and Table 4; the test statistic used is the Youden Index [15,16]. The graphs of survey results plotted against time shown in Figure 1 were generated in Excel. Survey dates were given by month and year and were coded as months with January 1997 = 1. Where two or more surveys were in the same month for the same country, they were separated by 0.05 months to allow Excel to plot the series shown in Figure 1. Analyses were unweighted (by sample size), as a majority of sample sizes were not recorded, and those reported were in a narrow range.

**Table 1:** Under-5 mortality rates (deaths/10,000/day) and GAM prevalence by livelihood and refugee status in Ethiopia, Kenya, Sudan and Uganda between 1997 and 2009 (n = number of survey results).

	Displaced persons (n) ^c,i^	Local (Resident) (n) ^d,j^	Mixed (n) ^e,k^
*A: Under-5 Mortality*			
Pastoralists ^a^	1.43 (89)	1.53 (186)	1.48 (138)
Agriculturalists ^b^	1.41 (39)	0.93 (315)	
Total ^f^	1.42 (128)	1.15 (501)	
*B: GAM (%)*			
Pastoralists ^g^	16.4 (173)	19.3 (302)	18.0 (188)
Agriculturalists ^h^	7.2 (104)	9.0 (389)	
Total ^l^	13.0 (277)	13.5 (691)	

Abbreviations: GAM, global acute malnutrition. The following refer to Mann-Whitney U tests. ^a^ Pastoralists population, comparing displaced persons, resident, and mixed groups: *p* = 0.742, n = 413; ^b^ Agriculturalist population, comparing displaced persons and resident groups: *p* = 0.003, n = 354; ^c^ Displaced population, comparing U5MR for pastoralists and agriculturalists: *p* = 0.934, n = 128; ^d^ Local (resident) population, comparing U5MR for pastoralists and agriculturalists: *p* = 0.000, n = 501; ^e^ Mixed not included for agriculturalists because n = 14; ^f^ Total, comparing refugees with local: *p* = 0.009, n = 629; ^g^ Pastoralists population, comparing displaced persons, resident, and mixed groups: *p* = 0.000, n = 663; ^h^ Agriculturalist population, comparing displaced persons and resident groups: *p* = 0.103, n = 493; ^i^ Displaced population, comparing GAM for pastoralists and agriculturalists: *p* = 0.000, n = 277; ^j^ Local (resident) population, comparing GAM for pastoralists and agriculturalists: *p* = 0.000, n = 691; ^k^ Mixed not included for agriculturalists because n=18; ^l^ Total, comparing displaced with local: *p* = 0.312, n = 968.

**Table 2:** Under-5 mortality (deaths/10,000/day) and GAM (%) by periods of years refugees/IDP, resident and mixed populations combined between 1997 and 2009 (n = number of survey results; *p* values estimated by Kruskall-Wallis non-parametric tests).

	Under-5 Mortality (n)	Global Acute Malnutrition (with oedema included) (n)
*A: Sudan (pastoralists)*		
1997–2002	1.83 (57)	18.9 (117)
2003–2006	1.40 (158)	17.7 (222)
2007–2009	1.09 (77)	17.4 (97)
Total	1.46 (292)	18.0 (436)
*p*	0.000	0.60
*B: Kenya (pastoralists)*		
1997–2004	1.90 (21)	17.9 (51)
2005–2009	0.87 (47)	18.6 (74)
Total	1.19 (68)	18.3 (125)
*p*	0.000	0.58
*C: Ethiopia (agriculturalists only)*		
1997–2002	1.59 (32)	9.2 (110)
2003–2006	0.94 (221)	9.3 (225)
2007–2009	0.56 (76)	8.8 (77)
Total	0.92 (329)	9.2 (412)
*p*	0.000	0.12
*D: Ethiopia (pastoralists only)*		
1997–2004	2.25 (30)	20.9 (65)
2005–2009	2.10 (33)	16.0 (36)
Total	2.18 (63)	19.1 (101)
*p*	0.39	0.10

**Table 3:** Under-5 mortality (deaths/10,000/day) and GAM (%) by period for refugees/IDP, resident and mixed populations in pastoralists in Sudan and Kenya between 1997 and 2009 (n = number of survey results; *p* values estimated by Kruskall-Wallis non-parametric tests).

	*U5MR, deaths/10,000/day*			*GAM %*		
*Time period*	*Displaced persons*	*Local (Resident)*	*Mixed*	*Displaced persons*	*Local (Resident)*	*Mixed*
1997–2002	1.77 (15)	1.95 (18)	1.84 (34)	15.6 (52)	20.9 (52)	19.5 (50)
2003–2006	1.58 (49)	1.28 (68)	1.41 (61)	18.1 (65)	19.7 (111)	16.4 (86)
2007–2009	1.09 (26)	0.88 (50)	1.10 (38)	15.5 (43)	18.2 (59)	16.8 (42)
Total	1.47 (90)	1.22 (136)	1.43 (133)	16.6 (160)	19.6 (222)	17.4 (178)
*p*	0.08	0.00	0.01	0.02	0.15	0.17

Note: for U5MR slopes over time are not significantly different between displaced persons and local population.

Table 4Example of correspondence between U5MR and GAM for surveys between 1997 and 2009.

**Table:** 

A: Kenya (pastoralists) at cut-off point of 20% GAM ^a^			
	**U5MR**		
**GAM**	≥2.0	<2.0	Total
≥20%	11	18	29
<20%	2	37	39
Total	13	55	68

GAM, global acute malnutrition; PPV, positive predictive value; Se, sensitivity; Sp, specificity, U5MR, under-5 mortality rate (deaths/10,000/day). ^a^ Se = 11/18 = 0.61, Sp = 37/55 = 0.67, PPV = 11/29 = 0.38, Se + Sp = 1.28.

**Table:** 

B: Ethiopia (agriculturalists only) at cut-off point of 10% GAM ^b^			
	**U5MR**		
**GAM**	≥2.0	<2.0	Total
≥10%	16	99	115
<10%	12	202	214
Total	28	301	329

GAM, global acute malnutrition; PPV, positive predictive value; Se, sensitivity; Sp, specificity, U5MR, under-5 mortality rate (deaths/10,000/day). ^b^ Se = 16/28 = 0.57, Sp = 202/301 = 0.67 , PPV = 16/115 = 0.13, Se + Sp = 1.24.

**Figure 1.** Patterns of survey results: malnutrition (GAM) and child mortality (U5MR) plotted by month of survey.

**Figure:**
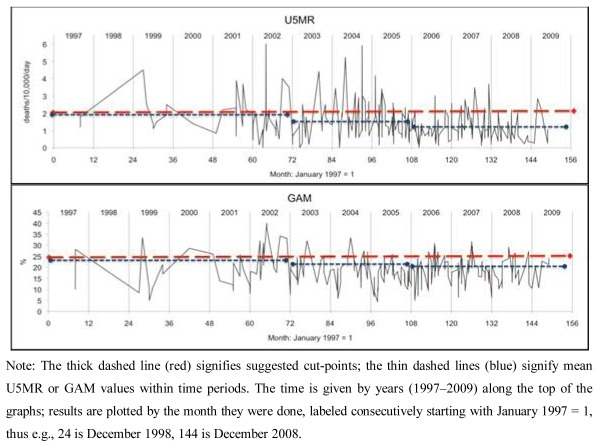
A: Sudan (pastoralists)

**Figure:**
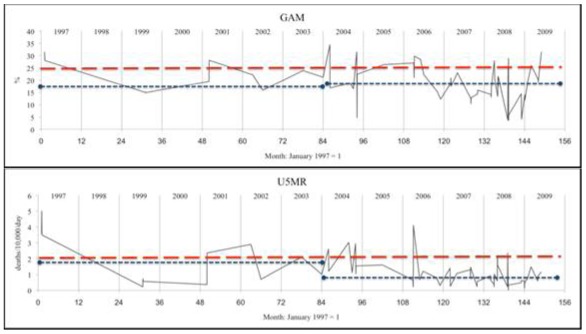
B: Kenya (pastoralists)

**Figure:**
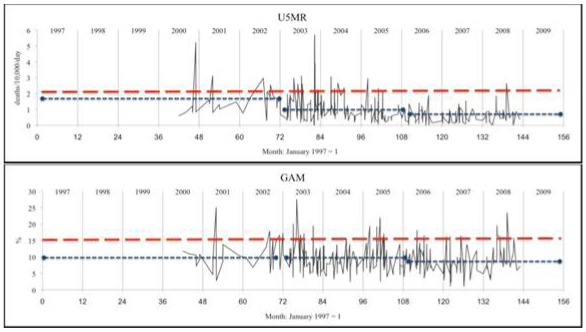
C: Ethiopia (agriculturalists only)

## 3. Results

### 3.1. Trends in GAM and U5MR

Analysis of 1,175 survey results from Ethiopia, Kenya, Sudan and Uganda for 1997 to 2009, showed that the average U5MR amongst displaced persons, the local population, and mixed groups was 1.15–1.42 deaths/10,000/day, as shown in Table 1A. Resident agriculturalists’ U5MR of 0.93 deaths/10,000/day was significantly lower (*p* < 0.005) than other groups, which experienced 1.1–1.53 deaths/10,000/day; the interaction for U5MR between displaced/local and livelihood was significant (*p* < 0.05). Thus the effect of displacement was significantly worse for agriculturalists children’s survival than for pastoralists.

IDPs had somewhat higher GAM (15.1%) compared to refugees (12.0%, *p* = 0.000). Grouping these together as displaced, Table 1B shows GAM prevalence remaining relatively similar within livelihood groups, across displaced persons, local and mixed populations; GAM however varies by a factor of two between pastoralist and agriculturalist populations. In displaced populations GAM was 16.5% in pastoralists compared with 7.2% in agriculturalists, and the difference is similar to the local populations (18.3% *vs*. 9.0%). It was not possible to compare U5MR and GAM within mixed population groups due to small sample sizes. While many surveys only estimated GAM and not U5MR, the GAM results remain similar when selecting the smaller number of surveys in which both U5MR and GAM were estimated. 

U5MR and GAM as individual survey results are plotted through time in Figure 1A–C for pastoralist populations in Sudan and Kenya and agriculturalist populations in Ethiopia. The graphics showing survey results plotted through time display variations and possible relationships between U5MR and GAM through time, showing substantial correspondence (e.g., when GAM is raised under-5 mortality is generally raised as well). Pastoralist populations in Sudan and Kenya have on average higher rates of U5MR and higher GAM than agriculturalists. 

Data on U5MR and GAM were averaged by time period as shown in Table 2A–C, and indicated by dashed horizontal lines in Figure 1A–C. U5MR fell considerably (40–65%) in all three situations, while average GAM showed little change, remaining at approximately 18% in Sudan and Kenya (pastoralist populations), and 9% in Ethiopia (agriculturalist). By the 2007–2009 time period, U5MR fell below 1 death/10,000/day in both Ethiopia (agriculturalists) and Kenya while their GAM prevalences changed relatively little. Average U5MR and GAM were also explored in the limited number of cases for Ethiopian pastoralists (Table 2D). Similar to Kenya, surveys were broken into two time periods (rather than three) due to smaller sample size. Unlike the other situations, in this group U5MR remained very high throughout this time, with GAM decreasing somewhat from 20.9% to 16.0%.

**Figure 2.** Associations of under-5 mortality (as log_10_ U5MR) with GAM for displaced persons, resident and mixed population combined by time periods (before 2005 and after), by livelihood, in Ethiopia, Kenya, Sudan, and Uganda.

**Figure:**
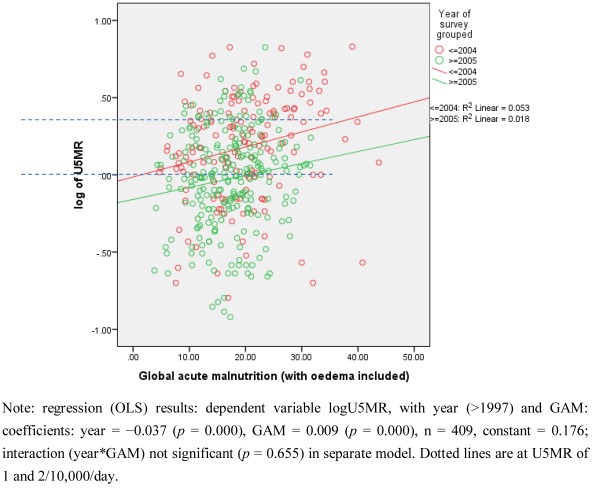
A: Pastoralists

**Figure:**
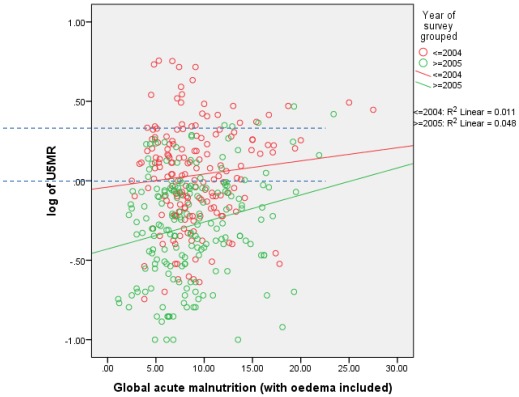
B: Agriculturalists

A comparison of trends between local residents and the displaced persons is shown in Table 3 for Sudan and Kenya data combined (to give a larger sample). For U5MR the falling trend is somewhat faster in local resident populations (but not significantly), and GAMs remain high with little trend in all groups.

### 3.2. Relationships of GAM with U5MR

GAM prevalence hardly changed through time while U5MR significantly decreased, except in the Ethiopian pastoralist group. Thus, the *relationship* *between* these two indicators appears to have changed over time. This is shown in Figure 2A and B. In both groups the U5MR at a given level of GAM fell significantly with time: as examples, for pastoralists U5MR was about 1.5/10,000/day at GAM of 20% in the period 1997–2005, but lowered to 1/10,000/day at this GAM level after 2005; for agriculturalists U5MRs were lower altogether, but at 5–10% GAM were 1/10,000/day prior to 2005, and only half that after 2005. Overall, the increase in U5MR per 10 percentage points increase in GAM was 1.2 to 1.4/10,000/day.

### 3.3. Agreement between GAM and U5MR

To investigate of the extent to which raised prevalences of GAM agree with elevated levels of U5MR, the discrimination (in terms of sensitivity and specificity) of GAM with different cut-points in identifying cases with U5MR greater than 1/10,000/day and 2/10,000/day were calculated. Illustrations of the calculation are given in Table 4A (Kenya) and Table 4B (Ethiopia, agriculturalists). For example, Table 3A shows that of the 13 cases with U5MR ≥ 2/10,000/day, 11 would be identified by GAM ≥ 20%, but at the expense of 18 false positives, with GAM ≥ 20% but U5MR < 2/10,000/day. 

One criterion for determining the most efficient cut-point is where sensitivity (Se) plus specificity (Sp) are maximized, calculated as (Se+Sp-1) (the Youden Index [15,16]). Results are summarized in Table 5. The value (Se+Sp-1) was highest at 20–25% GAM in Kenya and Sudan respectively (pastoralists), but at 10% in Ethiopia (agriculturalists). Within the Ethiopia pastoralist population, the index was highest at 15% GAM. The maximum value for the index was much the same for U5MR of both 1 and 2/10,000/day, and somewhat more efficient for the higher U5MR value.

**Table 5:** GAM cut-points associated with best identification of elevated U5MR of 1 and 2/10,000/day, estimated by sum of sensitivity plus specificity (minus 1).

Group	For U5MR > 1/10,000/day		For U5MR > 2/10,000/day		N
	GAM cut point %	(Se+Sp–1)	GAM cut point %	(Se+Sp–1)	
Sudan (pastoralists)	20%	0.20	25%	0.19	283
Kenya (pastoralists)	20%	0.43	20%	0.52	68
Ethiopia (agriculturalists)	10%	0.15	10%	0.24	329
Ethiopia (pastoralists)	15%	0.28	15%	0.37	62

**Figure 3:**
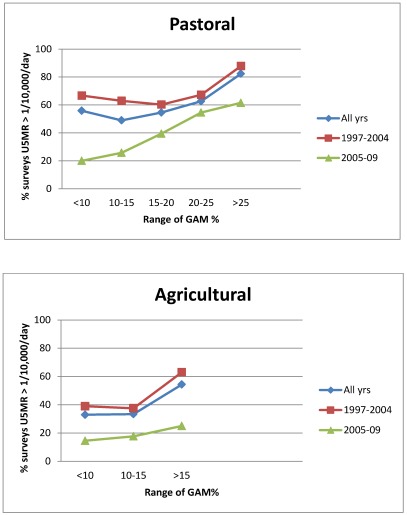
Percentage of surveys with U5MR > 1/10,000/day by range of GAM %, by livelihood group.

A question relevant to decision-making on needs for emergency intervention concerns when the U5MR is expected to be elevated; GAM is relevant when U5MR is not assessed (as in one third of the data here), and to add to the interpretation of new survey results when it is. The percentage of surveys with U5MR > 1/10,000/day by range of GAM (equivalent to the positive predictive value) is shown in Figure 3, separately for livelihood groups, and by time period cut at 2005, since the relation between GAM and U5MR was shown to change through time (see Figure 2). We can thus estimate the GAM at which the U5MR starts to rise. In the agricultural population, this is at 15% GAM or higher, more pronounced before 2005: thus for example over the whole time period, below 15% GAM 33% of surveys gave U5MR > 1/10,000/day, this rose to 55% after GAM of 15%. In the pastoral population over the whole period 50–60% of surveys with GAM less than 25% had elevated U5MR, and this rose sharply to over 80% above 25% GAM. In line with the observed decrease in U5MR (but not GAM) since 2005, the percentage of surveys with elevated U5MR is less in the more recent time period, for both livelihood groups, and the cut points less clear. In the earlier period there was less assistance (which is likely to account for at least part of the differences between periods), thus in a new situation before assistance is launched the earlier U5MR-GAM relation may be the more appropriate. Together with the results in Table 5, this suggests GAM cut-points of 10–15% for agricultural populations and 20–25% for pastoralists, for indicating likely sharp increases in U5MR, particularly when there is minimal assistance in place. The dotted horizontal lines in Figure 1A–C represents the higher levels of these suggested cut-points for GAM.

## 4. Discussion

### 4.1. GAM Prevalences Need Different Interpretation between Livelihood Groups

The major difference in GAM is between livelihood groups; pastoralists’ children having at least twice the GAM prevalence compared to those of agriculturalists (e.g., see Table 1). This difference was shown to be important in other data from this region [6], and reflects very different growth patterns in early childhood [5] and probably *in utero*. An important recommendation from this article is that analyses need to stratify by this factor. When GAM only is assessed, different prevalence cut-points for GAM by livelihood are needed to interpret U5MR risk, and hence the urgency and priority of interventions. When both U5MR and GAM are assessed, these results provide for some addition to the interpretation: knowledge of the usual associations can be taken into account.

The situation is complex in that mortality rates are higher in general for pastoralists than agriculturalists at all GAM levels (see Figure 3). If decisions on emergency interventions were based only on U5MR then pastoral populations would always take priority, even when GAM is relatively low. An issue here is to suggest how to decide when the data indicate unusual risk (*i.e.*, a potential emergency). 

Estimates of cut-points for deciding when unusual mortality risks are indicated as 20–25% GAM in pastoral populations and 10–15% GAM in agriculturalists. This difference in cut-points by livelihood is similar to but higher than those estimated in non-displaced populations in the region, where 15% and 10% cut-points, respectively for these groups were suggested [6]. The higher values here reflect on average more malnutrition and child mortality among these populations, but it is not suggested that the cut-points should differ between displaced and non-displaced populations. Operationally, cut-points should be specific to the populations affected in terms of livelihood, and resources available. 

Therefore, there seems no reason to perpetuate invariant GAM cut-points (as is the current practice), and some advantages in tailoring these to specific populations. Perhaps the main drawback of using one cut-point for all populations is to *underestimate* the increased risk to agricultural populations. As shown in Figure 3, these have much increased risk of raised U5MR at 15% GAM, at which point the U5MR risk is not raised above average for pastoralists. Thus, decisions to provide assistance based on the same GAM cut-points will cause under-response in agricultural populations and over-response in pastoral ones, if the aim is to prevent emergencies or unusually heightened risk.

### 4.2. U5MR: A Success Story?

U5MR fell substantially through the period (1997–2009) in populations surveyed in Sudan and Kenya and in Ethiopian agriculturalists, while GAM remained much the same (Table 2A–C). Mortality fell somewhat faster among the local population than among the displaced, although this was not significant (Table 3); GAM hardly altered in either group.

Improvement in U5MR has been seen in other studies among displaced populations in Africa. Salama *et al*. [17], noted that case-fatality rates had declined rapidly with better treatment—applying both to those in camps and others with access to humanitarian assistance—and that mortality was in fact higher at that time in the local (non-displaced) surrounding populations than in the displaced. In Darfur (Sudan), mortality fell while malnutrition remained high during 2003–2007 [18]. Prevalences of malnutrition among displaced or local populations have been reported elsewhere to persist at high levels and not to fall in a manner similar to mortality [19], as in our findings. 

Other studies have shown variable results. For example, the non-displaced population had higher GAM prevalences than displaced in Guinea-Bissau [20] and Congo [21], but not in Eastern Chad [22]. Thus the finding here of only small differences in GAM between displaced and local resident populations is not inconsistent with other results.

The substantial fall in mortality in these surveys may reflect the very extensive and increasing humanitarian assistance provided over the period studied. The plots in Figure 2 indicate that the improvement in U5MR occurred at all levels of GAM. The U5MR fall in the surrounding local resident population was similar to that for the displaced (Table 3). This could mean that assistance reached non-displaced people, a need recognized in the early 2000s [17]. It could also mean that mortality fell due to other factors affecting both population groups. While we do not have data on this, it does seem likely that the extensive assistance provided did have an impact on under-5 child mortality.

Once again the people of the Horn of Africa are suffering from severe food insecurity [1,2]. Financial and human resources are limited, and timely response is essential to save lives, especially those of children. At the same time, available data are difficult to unravel, covering different population groups, and generally result from *ad hoc* surveys. A first conclusion is that simply displaying the mortality and malnutrition indicators as plots through time, by livelihood group, helps greatly in visualizing the situation, its development, and how it currently compares with previous times. We suggest that data should routinely be presented by livelihood group and location, along the lines shown in Figure 1.

We also propose that response to the current severe food insecurity take into account differences between livelihoods—specifically disaggregating nutrition survey results between pastoralists and agriculturalists—and use population-specific cut-points to prioritize resource allocations and accordingly to make appropriate life-saving decisions. 

### 4.3. Limitations

The analyses reported here have certain limitations. The data are secondary, obtained from publications and databases that themselves have extracted results from survey reports. Moreover no check on quality was possible, although this is recognized to be highly variable [23]. However, this may be balanced out by random error. Analyses were unweighted (by sample size), but reanalysis for the minority of cases with known sample size showed no significant changes in results. A number of factors have been shown to correlate with mortality [24] including access to and quality of health care, food availability, and length of time people have spent in camps. None of these were available, and while malnutrition and mortality are causally related, this relation may be modified by such factors. The designation as displaced, local population, or mixed was usually reported in the survey data; where the delineation was unclear, the population would be defined as ‘mixed’. However, the livelihood status had to be inferred from the location and other descriptions of the population; this did not allow for closer definition when livelihoods combined cattle-raising and crop-growing (*i.e.*, agro-pastoralist). In cases where displacement was between pastoral and agricultural areas some classification error may have occurred. 

Severe acute malnutrition (SAM) was not studied extensively as an outcome indicator here, since only 6% of the cases showed prevalences raised above 5% (and lower values would represent few individual cases and be subject to wide confidence intervals). Thus, analyses using SAM in this dataset were not able to show useful associations. Nonetheless, the occurrence of SAM should be monitored from clinic or program data in a camp setting, where it is likely to be highly predictive of raised mortality [25].
